# Rationale for combination therapy of chronic myelogenous leukaemia with imatinib and irradiation or alkylating agents: implications for pretransplant conditioning

**DOI:** 10.1038/sj.bjc.6600242

**Published:** 2002-05-06

**Authors:** J Topaly, S Fruehauf, A D Ho, W J Zeller

**Affiliations:** Department of Internal Medicine V, University of Heidelberg, Hospitalstrasse 3, D-69115, Heidelberg, Germany; German Cancer Research Center (DKFZ), D0200, Im Neuenheimer Feld 280, D-69120, Heidelberg, Germany

**Keywords:** chronic myelogenous leukaemia, imatinib, STI571, Glivec, Gleevec, irradiation

## Abstract

The tyrosine kinase activity of the BCR–ABL oncoprotein results in reduced apoptosis and thus prolongs survival of chronic myelogenous leukaemia cells. The tyrosine kinase inhibitor imatinib (formerly STI571) was reported to selectively suppress the proliferation of BCR–ABL-positive cells. Assuming that imatinib could be included in pretransplantation conditioning therapies, we tested whether combinations of imatinib and γ-irradiation or alkylating agents such as busulfan or treosulfan would display synergistic activity in BCR–ABL-positive chronic myelogenous leukaemia BV173 and EM-3 cell lines. Further, primary cells of untreated chronic myelogenous leukaemia patients were assayed for colony forming ability under combination therapy with imatinib. Additionally, the cytotoxic effect of these combinations on BCR–ABL-negative cells was investigated. In the cell lines a tetrazolium based MTT assay was used to quantify growth inhibition after exposure to cytotoxic drugs alone or to combinations with imatinib. Irradiation was applied prior to exposure to imatinib. Interaction of drugs was analysed using the median-effect method of Chou and Talalay. The combination index was calculated according to the classic isobologram equation. The combination imatinib + γ-irradiation proved to be significantly synergistic over a broad range of cell growth inhibition levels in both BCR–ABL-positive cell lines and produced the strongest reduction in primary chronic myelogenous leukaemia colony-forming progenitor cells. Combinations of imatinib + busulfan and imatinib + treosulfan showed merely additive to antagonistic effects. Imatinib did not potentiate the effects of irradiation or cytotoxic agents in BCR–ABL-negative cells. Our data provide the basis to further develop imatinib-containing conditioning therapies for stem cell transplantation in chronic myelogenous leukaemia.

*British Journal of Cancer* (2002) **86**, 1487–1493. DOI: 10.1038/sj/bjc/6600242 www.bjcancer.com

© 2002 Cancer Research UK

## 

Chronic myelogenous leukaemia (CML) is a clonal disorder of the pluripotent stem cell with involvement of all haemopoietic lineages and is characterised by preferential expansion of myeloid cells. On the cellular level, CML is distinguished by the Philadelphia chromosome (Nowell and Hungerford, 1960), an abnormally short chromosome 22, which is found in more than 90% of all cases of CML, arising from a reciprocal t(9;22) translocation (Rowley, 1973). The product of this translocation, a 210-kD BCR–ABL protein (p210^BCR-ABL^), is characterised by a constitutively enhanced tyrosine kinase activity as compared with that of the normal c-ABL protein (Konopka *et al*, 1984) and is known to interfere with a variety of cytoplasmatic and cytoskeletal signalling proteins and cascades (Faderl *et al*, 1999; Deininger *et al*, 2000), which eventually leads to inhibition of apoptosis. The inherent resistance of residual BCR–ABL-positive cells to cytotoxic therapy is a major impediment to long-term management of CML.

After a median duration of 2–6 years under conventional therapy, the initial indolent chronic phase of the disease is followed by the accelerated phase and terminal blast crisis, which results in the patient's death within 3–6 months (Kantarjian *et al*, 1998). Allogeneic stem cell transplantation (allo-SCT) still remains the only proven curative option and particularly younger patients can benefit from this procedure (Gratwohl *et al*, 1998).

For patients without a suitable donor, high-dose conditioning therapy followed by autologous SCT (auto-SCT) is an option and durable remissions can be achieved in patients transplanted in the early chronic phase (Carella *et al*, 1999). The value of auto-SCT as consolidative treatment in blast crisis patients or patients with BCR–ABL-positive leukaemia transplanted in remission is the subject of ongoing trials (Goekbuget *et al*, 2001). Detection of residual leukaemic cells after high-dose chemotherapy is a strong predictor for clinical relapse in BCR–ABL-positive disease (van Dongen *et al*, 1998). Strategies to improve the conditioning therapy may help to increase the remission rates.

One of the new and currently very promising therapeutic strategies for CML is the targeting of the BCR–ABL tyrosine kinase (Druker *et al*, 1996). Recently reported clinical trials with the Abl tyrosine kinase inhibitor imatinib in blast crisis (Druker *et al*, 2001a), interferon-refractory or -intolerant chronic phase (Druker *et al*, 2001b), and in accelerated phase CML patients (Talpaz *et al*, 2000) showed encouraging results.

Nevertheless, 60% of myeloid blast crisis and almost all lymphoid blast crisis patients eventually relapsed (Druker *et al*, 2001a). Relapses were also observed in the accelerated and even in the chronic phase of the disease (Gorre *et al*, 2001; Hochhaus *et al*, 2001). Combination of imatinib with established cytotoxic therapy especially for the control of minimal residual disease as can be achieved by marrow-ablative conditioning therapy may allow to further reduce the leukaemic cell pool.

## MATERIALS AND METHODS

### Cells

The BCR–ABL-positive BV173 (lymphatic blast crisis) and EM-3 (myeloid blast crisis) human cell lines and the BCR–ABL-negative KG1a and HL-60 human leukaemic cell lines were obtained from the German Collection of Microorganisms and Cell Cultures (Braunschweig, Germany). The BCR–ABL status of the cell lines was confirmed using reverse transcriptase-polymerase chain reaction (data not shown). Cells were grown in RPMI-1640 medium supplemented with 10% (BV173, HL-60 and EM-3) or with 20% (KG1a) heat inactivated foetal calf serum (FCS), 2 mM L-glutamine, and penicillin/streptomycin (Life Technologies GmbH, Eggenstein, Germany) at 37°C in a fully humidified atmosphere of 95% air and 5% CO_2_. Primary cells were obtained from two patients in chronic phase CML. Mononuclear cells were isolated by Ficoll gradient centrifugation (Biocoll separating solution, Biochrom KG, Berlin, Germany).

### Irradiation and alkylating agents

γ-irradiation, busulfan and treosulfan were chosen for combination treatment in this study. A ^138^Cs source at a dose-rate of 0.72 Gy min^−1^ was used for irradiation. The busulfan solution (Busulfex injection) was obtained from Orphan Medical, Inc., Minnetonka, MN, USA, in a 10 ml ampoule containing busulfan 60 mg, dimethylacetamide 3.333 ml and polyethylene glycole 6.667 ml. Treosulfan (Ovastat 1000) was provided by Medac, Hamburg, Germany. Imatinib was provided by Novartis Pharma AG, Basel, Switzerland. Serial dilutions of the drugs were prepared by diluting the stock solutions in sterile Dulbecco's phosphate buffered saline (Dulbecco's PBS, Life Technologies GmbH) freshly prior to beginning of incubation.

Simultaneous exposure to imatinib in combination with busulfan or treosulfan was chosen to ensure a continuous inhibition of the BCR–ABL tyrosine kinase. Irradiation was applied prior to incubation with imatinib. Cells were either treated with six increasing doses (doubling with each increment) of busulfan, treosulfan, irradiation or imatinib alone or with the respective combinations. The doses for BV173 cells are given in Table 1

**Table 1:**
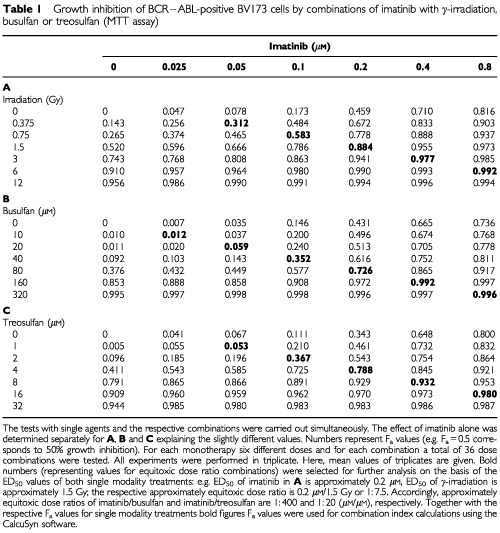
Growth inhibition of BCR–ABL-positive BV173 cells by combinations of imatinib with γ-irradiation, busulfan or treosulfan (MTT assay)

. For EM-3 cells following dose ranges were used: γ-irradiation 0.75–24 Gy, busulfan 5–160 μM, treosulfan 2–64 μM, and imatinib 0.025–0.8 μM.

### MTT assay

The MTT assay is based on the cleavage of the yellow tetrazolium salt 3-(4,5-dimethylthiazol-2-yl)-2,5-diphenyl tetrazolium bromide (MTT; SIGMA) to dark blue formazan crystals by viable cells (Mosmann, 1983). A high correlation between the viable cell number and formazan production has been reported (Finlay *et al*, 1986; Pieters *et al*, 1988). *In vitro* blast cell survival measured by the MTT assay correlates highly with blast cell proliferation measured by the classical ^3^H-thymidine incorporation assay (Norgaard *et al*, 1996). The MTT assay is widely used for assessment of combination therapies employing imatinib (Thiesing *et al*, 2000; Kano *et al*, 2001) and was performed as previously described (Topaly *et al*, 2001).

### Determination of Combination Index (CI)

**Figure 1:**
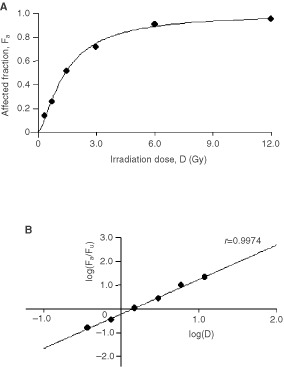
Example for the dose response curve of irradiation in BCR–ABL-positive BV173 cells (**A**) and the data transformation to allow calculation of the level of growth inhibition at a given dose according to the median effect equation of Chou and Talalay (**B**). The dose-response relationship is displayed as a linear regression line (*r* = 0.997). The *x*-intercept of the median effect-plot (**B**) represents the median effect dose (D_m_, ED_50_) at which a 50% growth inhibition (F_a_ = 0.5) occurs. The six data points in **A** and **B** correspond to the six original data points of irradiation alone in Table 1 (1st data column). *F_a_*, affected fraction; *F_u_*, unaffected fraction; *D*, dose.

Dose-effect relationships were analysed using the median-effect method (Chou and Talalay, 1984; Topaly *et al*, 2001), an example is given in Figure 1

. The combination index (CI) was used to express synergism (CI<1), additivity (CI=1) or antagonism (CI>1) and was calculated according to the classic isobologram equation:


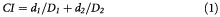


**Figure 2:**
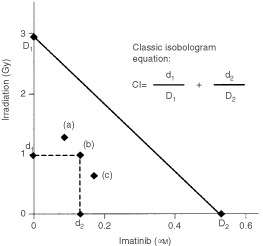
Example of a classic isobologram at ED_75_. Imatinib is combined with γ-irradiation in BV173 cells. The ED_75_ values were calculated from a series of primary data such as presented in Table 1 using CalcuSyn software. Means of three experiments were calculated and an isobologram was drawn by MS Excel. The three data points shown (**a**, **b**, and **c**) correspond to imatinib [μM]/γ-irradiation [Gy] ratios of 1 : 15, 1 : 7.5 (approximately equitoxic combination), and 1 : 3.75, respectively. The corresponding CI values as calculated following the classic isobologram equation are 0.572, 0.583, and 0.593. As CI values of <1 express synergism, imatinib + γ-rradiation represents an example of a strongly synergistic combination.

**Figure 3:**
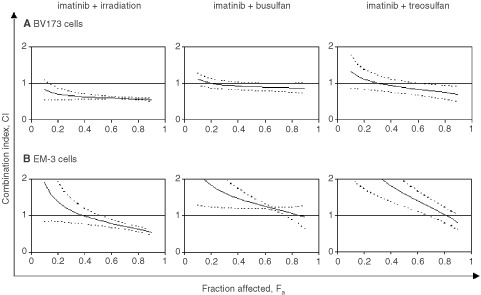
Combination index (CI) as a function of affected fraction (F_a_) in BCR–ABL-positive BV173 (**A**) and EM-3 (**B**) cells. F_a_ values of e.g. 0.25, 0.5 or 0.75 correspond to 25, 50 or 75% growth inhibition. CI values were calculated for distinct F_a_ values (e.g. F_a_ 0.1, 0.2, etc.) on the basis of experimentally determined F_a_ data such as shown in Table 1. By this method, CI values for different levels of growth inhibition and different drug combinations can easily be compared. The synergistic activity increases with higher levels of growth inhibition in all combinations (most apparently in the combination γ-irradiation + imatinib) suggesting optimal (maximal) activity of the pro-apoptotic treatment (γ-irradiation, busulfan, treosulfan) when imatinib is efficiently blocking BCR–ABL tyrosine kinase activity and the associated anti-apoptotic mechanisms. *Solid lines*, means; *dotted lines*, 1 s.d.-range.

In this equation, *D_1_* and *D_2_* represent the doses of drug 1 and drug 2 alone, required to produce x% effect, and *d_1_* and *d_2_* are the doses of drugs 1 and 2 in combination required to produce the same effect. Figure 2

illustrates this equation using three different dose ratio combinations of the two treatment modalities, each effecting 75% growth inhibition (ED_75_). Since different CI values can be observed at different levels of growth inhibition (fraction affected, F_a_), presentation of data in CI *vs* affected fraction (F_a_) plots is reasonable (Figure 3

). CI values were initially calculated for three separate experiments using CalcuSyn Software and then transferred to MS Excel for calculation of mean values and standard deviation (s.d.); F_a_-CI plots were drawn using MS Excel.

### CFC Assay

4×10^4^ (patient 1) or 2×10^5^ cells (patient 2), cell numbers producing 50–70 colony-forming units granulocyte/macrophage (CFU–GM) per plate in untreated controls, were plated in duplicate in MethoCult GF H4434 methylcellulose (MC; Stem Cell Technologies, Vancouver, Canada) containing SCF (50 ng ml^−1^), GM–CSF (10 ng ml^−1^), IL-3 (10 ng ml^−1^), and erythropoetin (3 units ml^−1^) and incubated for 14 days at 37°C in humidified atmosphere containing 5% CO_2_ as previously described (Kasper *et al*, 1999). Individual CFU-GMs were counted. Mean values of duplicates for each patient were used for further analysis. Doses of the drugs and γ-irradiation used in CFC assay are given in Figure 5.

### Apoptosis assay

Annexin V/propidium iodide apoptosis assay was performed using the Annexin V-FITC Kit (Immunotech, Marseille, France) 24 h after beginning of incubation of EM-3 cells according to the manufacturer's protocol. The data were collected and analysed with a FACScalibur flow cytometer (Becton Dickinson, Heidelberg, Germany) using the CellQuest Software (Becton Dickinson).

### Statistics

Results are shown as mean values±standard deviation (s.d.) of three separate experiments. Statistical significance of the data was calculated by Student *t*-test. A significance level of *P*<0.05 was chosen.

## RESULTS

### Single agent therapy

ED_50_ values of imatinib alone were assessed following 48 h of incubation for all cell lines. The BCR–ABL-positive cell lines BV173 and EM3 were highly susceptible to imatinib with ED_50_ values of 0.30±0.08 μM (mean±s.d., *n*=9) and 0.11±0.03 μM (*n*=3), respectively. The ED_50_ values in BCR–ABL-negative cell lines HL-60 and KG1a were 17.1±0.7 μM and 26.7±3.3 μM, respectively. The therapeutic index of imatinib *in vitro*, defined as the ratio of ED_50_ in BCR–ABL-negative cells (dose-limiting side effect) to ED_50_ in BCR–ABL-positive cells (therapeutic effect), ranged from 57 to 243. For assessment of combination effects in BCR–ABL-negative cells a dose of 1.0 μM imatinib was used since this concentration failed to measurably affect their growth while causing more than 80% of growth inhibition in BV173 and EM-3 cells.

The sensitivity of cells to busulfan was relatively uniform ranging from an ED_50_ of 56.2±12.4 μM in HL-60 to 90.8±11.3 μM in BV173 cells. On the contrary, the sensitivity to γ-irradiation varied widely. BV173 cells proved to be most sensitive (ED_50_=1.70±0.95 Gy) while in EM-3 cells the ED_50_ was 6.39±0.69 Gy; in KG1a cells 10 Gy produced only minor growth inhibition of less than 20%. The ED_50_ of treosulfan ranged from 5.03±0.24 μM in BV173 cells to 54.4±3.7 μM in KG1a cells.

In all experiments the linear correlation coefficient of the median-effect plot (*r)* was >0.96 providing a reliable basis for further calculations.

### Combination treatment of BCR–ABL-positive cells

Therapeutic effects of approximately equitoxic molar ratios were chosen for the assessment of the combination index (CI). Since each cell line displayed different sensitivity to single modality treatments tested, the equitoxic molar ratios were adjusted individually for each BCR–ABL-positive cell line (bold numbers in Table 1). An example of the experimental design is given in Table 1.

Both in BV173 and in EM-3 cells significant synergistic effects (*P*<0.05) of imatinib + γ-irradiation were observed. In BV173 cells, the synergistic effect became apparent already at inhibition levels above 30% (F_a_=0.3) and still increased at higher inhibition levels. In combinations with busulfan or treosulfan, only additive effects were observed (Figure 3A).

In EM-3 cells, higher CI values were generally observed with all combinations tested. A synergism of imatinib + γ-irradiation became apparent at higher levels of growth inhibition than in BV173 cells (Figure 3B). Combinations with busulfan and treosulfan were again additive or even slightly antagonistic (Figure 3B).

**Figure 4:**
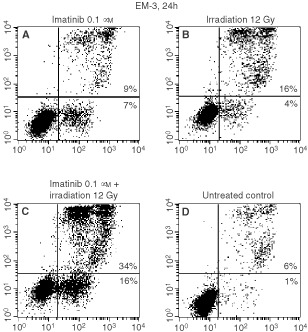
Apoptosis in EM-3 cells 24 h after start of incubation as detected by the annexin V/propidium iodide assay. Compared to treatment with imatinib alone or γ-irradiation alone (**A**, imatinib; **B**, γ-irradiation) a striking increase in the early apoptotic (lower right quadrant: annexin V+/PI−) and dead cell (upper right quadrant: annexin V+/PI+) fractions was observed (**C**) when the cells were treated with the combination. **D**, untreated cells.

The annexin V/propidium iodide apoptosis assay showed a considerable increase of the apoptotic cell fraction in EM-3 cells treated with imatinib + γ-irradiation as compared to treatment with imatinib or γ-irradiation alone (Figure 4

). This finding confirms that the observed synergism is based on an pro-apoptotic mechanism of action.

**Figure 5:**
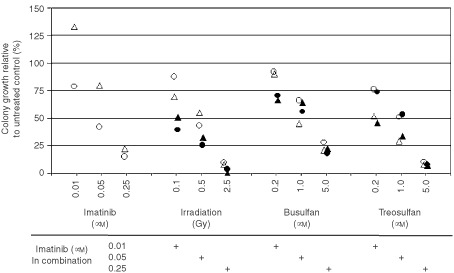
Activity of imatinib combinations in primary CML progenitor cells obtained from two patients. Imatinib + γ-irradiation produced a stronger growth inhibition than either treatment alone over the whole dose range tested as opposed to imatinib + busulfan and imatinib + treosulfan. *Triangles*, patient 1; *circles*, patient 2; *open symbols*, monotherapy; *filled symbols*, combination with imatinib.

In CFC assays with primary CML progenitor cells combination of imatinib and γ-irradiation produced a stronger growth inhibition than either treatment alone over the whole dose range tested (Figure 5

). Combinations of imatinib + busulfan and imatinib + treosulfan were inferior.

### Combination treatment of BCR–ABL-negative cells

**Figure 6:**
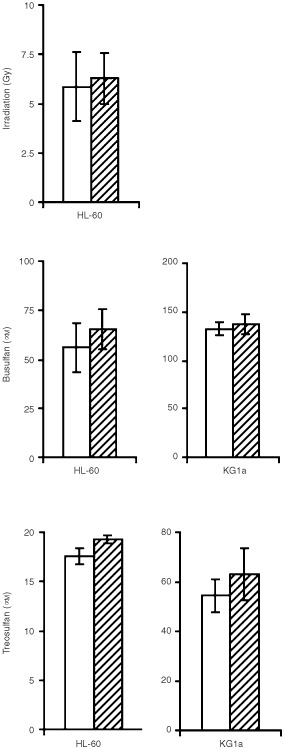
Effects of γ-irradiation, busulfan or treosulfan (each±1.0 μM imatinib) on BCR–ABL-negative cells: ED_50_ values of γ-irradiation, busulfan or treosulfan alone and the respective ED_50_ values in combination with imatinib do not differ significantly. Addition of imatinib does not increase the cytotoxicity on BCR–ABL-negative cells. *White columns*, cytotoxic treatment alone; *hatched columns*, respective combination with imatinib; *bars*, 1 s.d. Means of three paired experiments are presented (*P*>0.05).

Since imatinib itself had no measurable effect on BCR–ABL-negative cells at concentrations up to 1.0 μM, the median-effect method could not be applied to assess combination effects. Therefore, ED_50_ values of γ-irradiation, busulfan or treosulfan alone were compared with the respective ED_50_ values in the presence of 1.0 μM imatinib. As shown in Figure 6

, no increase of γ-irradiation-, busulfan- or treosulfan-induced cytotoxicity by imatinib was observed; the respective ED_50_ values were even slightly higher when combined with imatinib (not significant).

## DISCUSSION

This study shows a strong synergism of the combination of imatinib + γ-irradiation in BCR–ABL-positive lymphoid and myeloid blast crisis cells and its superiority to combinations of imatinib with the alkylating agents busulfan or treosulfan which were merely additive. Imatinib + γ-irradiation was also the most active combination against primary colony-forming CML cells. In BCR–ABL+ cell lines, doses chosen (with the exception of busulfan) came close to the clinically relevant dose range; in primary CML progenitors we chose a dose range of 0.1–2.5 Gy γ-irradiation and 0.2–5.0 μM busulfan or treosulfan (Figure 5) which would be feasible to be used *in vivo* (Körbling *et al*, 1986; Jacobson *et al*, 2001; Scheulen *et al*, 2000). Synergistic effects of imatinib with γ-irradiation and chemotherapeutic agents in leukaemic cell lines correlated well with the effects in primary CML cells.

*In-vitro* combination treatment data have already contributed to the design of current clinical trials with imatinib (Fruehauf and Hochhaus, 2001; Druker *et al*, 2001c). Consideration of the present results for clinical pretransplant conditioning therapy of CML patients should be discussed.

Since imatinib alone has little or no measurable effect and does not potentiate the effects of established cytotoxic treatment in BCR–ABL-negative cells at therapeutically relevant concentrations, even merely additively acting imatinib-containing combinations as shown in CML cells should exert more selective toxicity on the leukaemic cell clone as compared with either chemo- or radiotherapy alone. Synergistic combinations exert this effect even stronger.

Total body irradiation (TBI) or busulfan are contained in marrow ablative conditioning regimens and treosulfan (Loeb, 1964) is being tested in clinical trials in toxicity-reduced conditioning (Casper *et al*, 2000; Ploemacher *et al*, 2000). TBI/cyclophosphamide and busulfan/cyclophosphamide regimens are used alternatively for pretransplant conditioning therapy. A synergism between imatinib and mafosfamide, an active form of cyclophosphamide representing an essential part of the conditioning therapy, has been shown previously (Topaly *et al*, 2001). It is controversial which conditioning regimen, TBI/cyclophosphamide or busulfan/cyclophosphamide, should be preferred for conditioning in the clinical setting. For treatment of BCR–ABL-positive acute lymphoblastic leukaemia or lymphatic blast crisis of CML, TBI-containing regimens are generally preferred due to the higher irradiation sensitivity of lymphoid blasts and due to the risk of relapse in the central nervous system (Granados *et al*, 2000). In the myeloid blast crisis the choice of regimen is less uniform. Although TBI-containing conditioning seems to be more toxic as compared to its busulfan-containing counterpart, the speed of engraftment, event-free survival and relapse rate are not significantly different (Clift *et al*, 1994). We hypothesise that in the autologous (Pigneux *et al*, 1999) and possibly in the allogeneic transplantation setting TBI/cyclophosphamide + imatinib based on their synergistic effects could produce superior disease-free survival, even though risks and benefits should be thoroughly evaluated in future clinical trials. Treosulfan is employed alternatively to busulfan in conditioning regimens (Casper *et al*, 2000) and could also be supplemented with imatinib in CML patients. As a next step of preclinical testing, these combinations can be assessed in a ‘mock-conditioning’ scenario, i.e. in a mixed population of BCR–ABL-positive and -negative cells.

In conclusion, this is the first report on synergistic interaction between imatinib and γ-irradiation. It highlights the potential of combination treatment of CML employing specific BCR–ABL tyrosine kinase targeting and provides a rationale for the design of future conditioning protocols for stem cell transplantation in CML.
